# Impact of First SARS‐CoV‐2 Infection Variant on Serological Responses Against Omicron: Findings From the SIREN Study

**DOI:** 10.1111/irv.70204

**Published:** 2026-02-22

**Authors:** Ferdinando Insalata, Ana Atti, Edward J. Carr, Ashley D. Otter, SIREN study group, Sarah Foulkes, Giulia Dowgier, Agnieszka Hobbs, Mary Y. Wu, Susan Hopkins, Andre Charlett, Rupert Beale, Victoria Hall

**Affiliations:** ^1^ Antimicrobial Resistance and Healthcare Associated Infections Division United Kingdom Health Security Agency (UKHSA) London UK; ^2^ Department of Mathematics Imperial College London London UK; ^3^ UCL Centre for Kidney & Bladder Health, Royal Free Hospital London UK; ^4^ The Francis Crick Institute London UK; ^5^ Emerging Pathogen Serology UK Health Security Agency (UKHSA) Salisbury UK; ^6^ COVID Surveillance Unit, The Francis Crick Institute London UK

**Keywords:** COVID‐19 serological testing, immunity, reinfection, SARS‐CoV‐2, SARS‐CoV‐2 variants

## Abstract

**Introduction:**

Despite the existing hybrid immunity, a sharp increase in SARS‐CoV‐2 reinfections was observed worldwide following Omicron variant emergence. We investigated whether the first infecting variant indelibly shapes serological responses against Omicron (BA.1 and BA.2) reinfection.

**Methods:**

Participants with a sequence‐confirmed Alpha (*n* = 23) or Delta (*n* = 10) first infection before third vaccine dose (V3) that subsequently had a BA.1 or BA.2 reinfection were selected. Sera were tested for anti‐SARS‐CoV‐2 spike (anti‐S) and live virus microneutralisation (LV‐N) against Ancestral, Alpha, Delta, Omicron BA.1 and BA.2. Antibody responses and waning post‐V3 were compared by first infection variant using mixed‐effect models, as well as inferred titres days before reinfections. Individual's neutralisation responses were compared 12 weeks post‐V3, among those with Alpha and Delta primary infection.

**Results:**

After V3, those with Delta first infection had higher LV‐N Omicron BA.1 titres (fold difference (FD) = 2.7, *p* = 0.05) compared to Alpha primary infection. Participants with Delta first infection presented higher LV‐N BA.1 (FD = 1.89, *p* = 0.004) and LV‐N BA.2 (FD = 2.06, *p* = 0.001) titres pre‐Omicron reinfections. Individuals' neutralisation responses against Ancestral were higher than any other subsequent variants, regardless of first infection variant.

**Conclusions:**

A previous Delta SARS‐CoV‐2 infection induced a higher serological response against a subsequent Omicron infection when compared to Alpha first infections.

## 1 Introduction

SARS‐CoV‐2 reinfections have been reported since 2020 and were considered a relatively rare event until the emergence of Omicron variants from November 2021, particularly BA.1 and BA.2 subvariants. Omicron had strong immune evasion potential due to a larger number of mutations in spike (S) compared to previous SARS‐CoV‐2 variants and was responsible for a remarkable increase in SARS‐CoV‐2 infection rates worldwide, despite most of the population being previously vaccinated, infected or both [1, 2, 3, 4]. As expected, the first generation of vaccines showed reduced effectiveness against Omicron. As reinfection became common, it became important to understand the nature and extent of protection offered by hybrid immunity conferred by both infection and vaccination. In particular, it is unclear to what extent the variant that an individual with hybrid immunity was first exposed to contributed to protection at the time of Omicron exposure [1, 2].

During the early stages of the pandemic, previous infection was associated with high protection against SARS‐CoV‐2 reinfection [5]. This persisted despite the rapid spread and rise in infection rates with the emergence of the more transmissible Alpha variant in the UK (December 2020) [6, 7]. Subsequently, the Delta variant has been shown to be even more transmissible than Alpha and rapidly became dominant in the United Kingdom by May 2021, although vaccination reduced the transmissibility of both variants [8]. A modest reduction in vaccine effectiveness was observed against the Delta variant compared to Alpha after two vaccine doses [9].

COVID‐19 vaccines, although effective in preventing severe disease for long time periods, offer only short‐term immunity against infection. This is due to the mismatch between vaccine antigen and emerging variants, and also due to waning in neutralising antibody titres after vaccination [10]. More substantial and longer‐lasting protection has been observed in those who have been both vaccinated and infected, compared to those vaccinated without previous infection. This is presumably due to greater antigen dose and longer expression [11, 12].

In this context, a key aspect to be considered is how the exposure to viral antigens may shape the subsequent protection to related strains—also known as immunological imprinting [13]. For SARS‐CoV‐2, this phenomenon can be induced both by infection and vaccination and, in some interpretations of imprinting, may interfere with developing a broader immune response against emerging variants [14]. It has been reported that individuals infected with a newer SARS‐CoV‐2 variant who were previously exposed to Ancestral SARS‐CoV‐2 antigens (deriving both from infection and/or vaccination) had a higher neutralisation response against Ancestral than the newer infection variant, which is sustained over time [15, 16, 17, 18, 19]. This could potentially explain the lower protection offered by vaccines against Omicron variants.

Within the SARS‐CoV‐2 Immunity and Reinfection Evaluation (SIREN) study, we aim to investigate how the first SARS‐CoV‐2 infection variant shapes the serological response against Omicron (BA.1 and BA.2) reinfection.

## 2 Materials and Methods

### 2.1 Study Population and Design

We conducted an analysis of sequential sera samples from a subset of SIREN participants, who regularly underwent SARS‐CoV‐2 PCR and serology testing (ISRCTN 11041050). The SIREN study protocol is described elsewhere [20].

### 2.2 Case and Sample Selection

We selected participants according to the criteria below:

- Inclusion criteria
- Had a sequence‐confirmed first infection by Alpha or Delta variant;
- Had a subsequent Omicron BA.1 or BA.2 infection;
- Had received a third vaccine dose (V3) before their Omicron reinfection;
- Had at least one serum sample available between V3 and their Omicron reinfection
- Exclusion criteria:
- Had received a fourth vaccine dose before their Omicron reinfection;
- Had positive anti‐SARS‐CoV‐2 nucleocapsid (anti‐N) before their first infection (likely previously infected)

### 2.3 Sample Testing

All sera samples available from the selected participants were tested for anti‐N antibodies, anti‐SARS‐CoV‐2 spike (anti‐S) antibodies and live virus microneutralisation (LV‐N) against Ancestral, Alpha, Delta, BA.1 and BA.2 variants. Details on antibody testing are provided elsewhere [21, 22]. The LV‐N quantitative range is 40–2560 and, for the purpose of this analysis, titres above the quantitative range were replaced with the value at the top of the quantitative range (2560). For sera whose dilution did not cross 50% inhibitory concentration, we qualitatively reported as no neutralisation, weak neutralisation or complete neutralisation, assigning values of 5, 10 and 5120, respectively, for visualisation. When calculating geometric means, we moved values outside of the quantitative range to the endpoints.

### 2.4 Data Analysis and Modelling

#### 2.4.1 Dynamics of Antibody Levels and nAbs Titres Post‐V3 by Variant of First Infection

We compared peak antibody responses and antibody waning following V3 by variant of first infection (Alpha and Delta). We reported the fold difference (FD) as being the ratio of geometric means between the two groups defined by the first‐infection variant. All sera samples taken from 7 days after the third vaccine dose to assess peak antibody response, consistent with previous studies, and until 7 days before reinfection were included [22].

Trajectories (logarithmic scale) of antibody metrics were modelled using mixed‐effect models, as random intercepts at participant level were supported for all antibody responses (likelihood ratio test); for anti‐S, random slopes were also supported. Time since third dose and variant of first infection were included as predictors. Differences in peak values (models intercepts) were assessed using Wald test, allowing for different decay rates by variant of first infection via an interaction term between the variant and time variable. Overall decay rates were estimated by fitting model without the interaction term. We also controlled for time since first infection, including the period between first infection and third vaccine dose, assessing its significance via Wald test. For anti‐S titres, a standard linear mixed model was used; for nAb titres, mixed effect Tobit model was applied to account for left and right censoring of titres falling outside the quantitative range of the assay.

#### 2.4.2 Comparing Inferred Antibody Response at Time of BA.1 Infection by Variant of First Infection

We compared inferred antibody responses by variant of first infection at the time of BA.1 infections. Fitted models were used to predict the antibody levels and nAb titres at this specific time. All participants were included in this analysis. For people who were infected with BA.1, we inferred at 7 days before the PCR confirming reinfection. For people who were not infected with BA.1 (but later with BA.2), we matched time since third dose: We predicted levels/titres at 12 weeks after third dose, 7 days before the median time of a BA.1 infection (which is 13 weeks after third dose). On these inferred values, we assessed differences by variant of first infection by using *t*‐test with unequal variances (Welch's test), as the spread of data points in the groups compared was visibly different in most cases. We replaced values outside the quantitative range (full neutralisation) with the upper end of the quantitative range. On the same inferred levels and titres, we assessed differences by sub‐variant of reinfection, using the same statistical test.

#### 2.4.3 Comparing Inferred Antibody Response Immediately Before BA.2 Infection by Variant of First Infection

We compared predicted antibody responses by variant of first infection, immediately before BA.2 reinfection. Only BA. 2‐infected participants were included in this analysis to have comparable decay times since V3. Antibody levels and nAb titres of participants with BA.2 infection were inferred at 7 days before reinfection. We tested for differences in geometric means by variant of first infection across anti‐S and different nAb titres using Welch's test.

#### 2.4.4 Comparing Neutralisation Responses Among Those With Alpha or Delta First Infection

We compared inferred individuals' neutralisation responses against LV‐N Ancestral, LV‐N Alpha, LV‐Delta, LV‐N Omicron BA.1 and LV‐N Omicron among those who had Alpha first infection or Delta first infection at 12 weeks after V3 (proxy time of BA.1 infections). We reported the fold change (FC) as being the ratio of the values of different responses (e.g., LV‐N Alpha to LV‐N Delta) in a participant. All participants were included in this analysis. We used the same method to infer titres at this specific time as described above. We calculated geometric means of LV‐N Ancestral, LV‐Alpha, LV‐Delta, LV‐N Omicron BA.1 and LV‐N Omicron inferred titres.

## 3 Results

A total of 87 samples from 33 participants were selected. Samples taken between 8 October 2021 and 7 April 2022 were included in the analysis.

**FIGURE 1:**
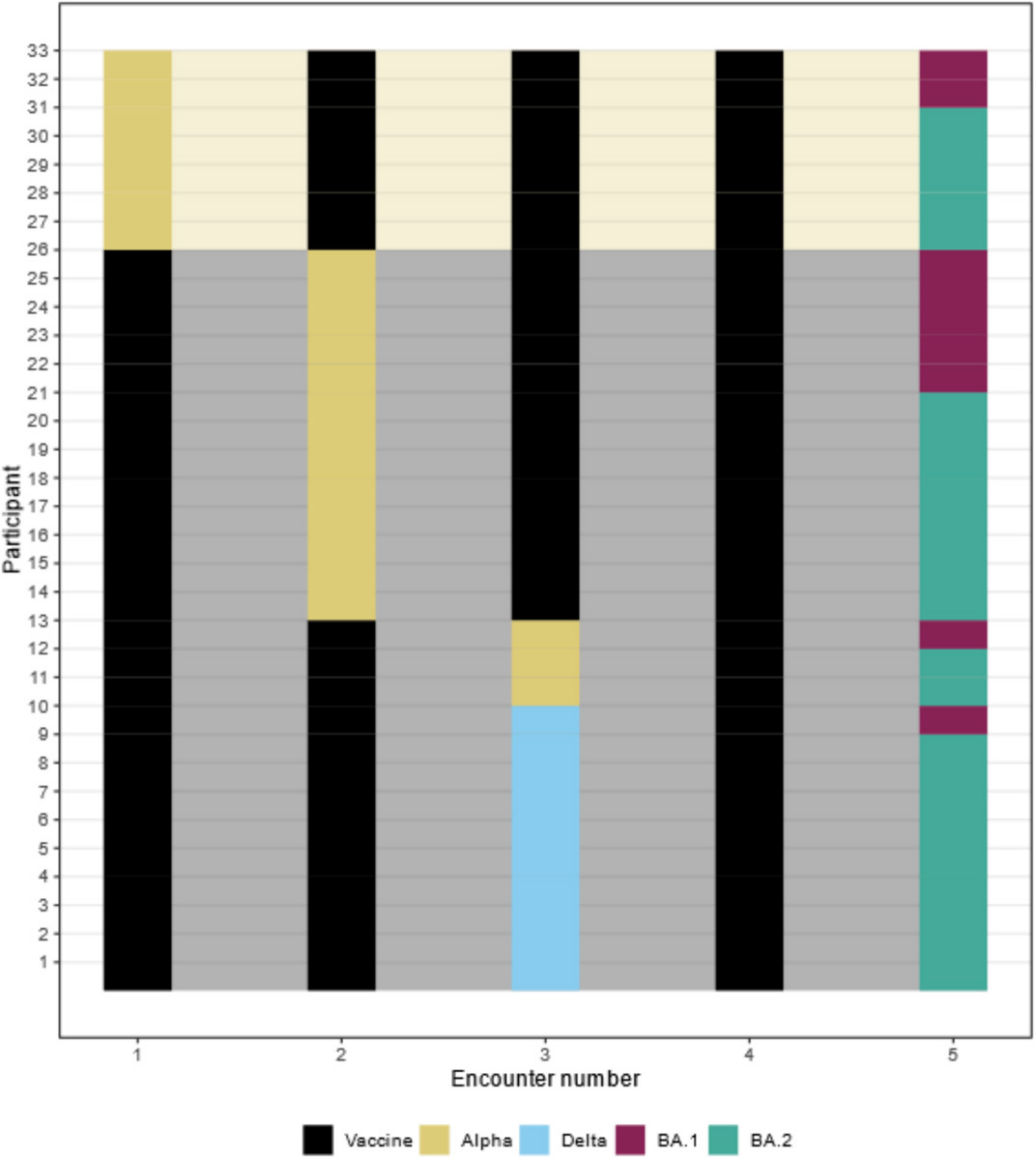
Antigenic encounters for each participant. Each line represents one participant included in this analysis. For example, Participant 13 had their first vaccine dose, followed by an Alpha first infection; after that, they had their second and third vaccine dose, followed by Omicron BA.2 infection.

Selected individuals were predominantly female (82%) and of white ethnicity (88%), with a median age of 44 years (interquartile range (IQR) 34–52) (Table 1). First infections occurred between 29 December 2020 and 5 November 2021; 70% (23/33) were Alpha and 30% (10/33) were Delta. A comparison on demographics between participants with Alpha and Delta first infection is reported in Supplementary Table S1. Patterns of antigenic exposures from all participants are described in Figure 1.

**TABLE 1:** Demographics of the re‐infection study compared to the whole of the SIREN study. The individuals selected for this study (*n* = 33) are also included in the whole SIREN cohort (*n* = 44,126).

Characteristic	Re‐infection, *N* = 33^a^	SIREN cohort, *N* = 44,126^a^
Sex		
Female	27 (82%)	36,849 (84%)
Male	6 (18%)	7212 (16%)
Non_binary	0 (0%)	33 (< 0.1%)
Prefer_not	0 (0%)	32 (< 0.1%)
Age		
Under 25	1 (3.0%)	1764 (4.0%)
25 to 34	9 (27%)	9030 (20%)
35 to 44	7 (21%)	10,871 (25%)
45 to 54	12 (36%)	13,169 (30%)
Over 55	4 (12%)	9292 (21%)
Ethnic group		
White	29 (88%)	38,678 (88%)
Asian	3 (9.1%)	3237 (7.3%)
Black	1 (3.0%)	885 (2.0%)
Mixed race	0 (0%)	680 (1.5%)
Other ethnic group	0 (0%)	557 (1.3%)
Prefer not to say	0 (0%)	89 (0.2%)
Patient facing		
Yes	27 (82%)	37,856 (86%)
No	6 (18%)	6270 (14%)
Comorbidities		
No medical condition	22 (67%)	33,018 (75%)
Chronic respiratory conditions	8 (24%)	5460 (12%)
Chronic non respiratory conditions	3 (9%)	4677 (10%)
Immunosuppression	0 (0%)	971 (2.2%)

^a^ *n* (%).

Reinfections occurred between 20 December 2021 until 21 June 2022; 27% (9/33) of participants were reinfected with BA.1 and 73% (24/33) with BA.2. A comparison of demographics between participants with BA.1 and BA.2 reinfections is shown in Supplementary Table S2.

The median time between first infection and reinfections was 382 days (IQR 236–424). The median interval between first infection and reinfection for those with Alpha first infection was 417 days and 198 days for those with Delta first infection, which was significantly shorter (Brown–Mood median test, *p* < 0.001). The median time between third vaccine dose and reinfection was 140 days (IQR 101–165) and was comparable between Alpha and Delta first‐infected groups (median 140 vs. 142 days, respectively; Brown–Mood median test, *p* = 0.91) (Supplementary Figure S1).

Regarding vaccine manufacturers, 90% (30/33) of participants received BNT162b2 mRNA for their primary course (first two doses) and 10% (3/33) received ChAdOx adenoviral; as for boosters (V3), 89% (29/33) received BNT162b2 mRNA and 11% (4/33) received Moderna mRNA.

### 3.1 Dynamics of Antibody Levels and nAbs Titres Post‐V3 by Variant of First Infection

**FIGURE 2:**
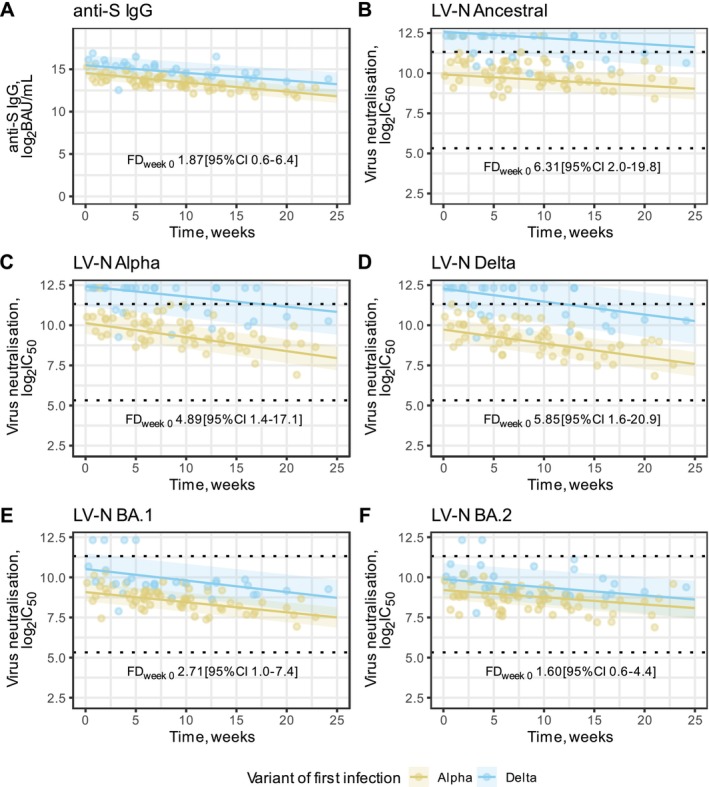
Estimated trajectories for anti‐S and neutralising antibodies by variant of first infection. (A) anti‐S IgG plotted against time since 1 week after each individual's third vaccine dose (time 0 = V3 + 7d) for participants with an Alpha or Delta first infection. (B–F) Live virus neutralisation (LV‐N) titres plotted against time since each individual's third vaccine dose (V3) for participants with an Alpha or Delta first infection. Ancestral, Alpha, Delta, Omicron BA.1 and Omicron BA.2 are shown in (B)–(F), respectively. Live virus neutralisation titres are reported as the reciprocal of the dilution at which 50% inhibition of viral entry occurred (IC_50_). In (A–F), all serological data are log2‐transformed and a population‐level fit line and confidence intervals are shown for Alpha or Delta first infections. Fold differences (FD) at week 0 of the model fits (V3 + 7d) between Alpha and Delta first infections are reported on a linear scale. In (B–F), dashed horizontal lines indicate the upper and lower bounds of the quantitative range.

We compared at peak response and decay rates by primary infection variant. For peak responses at 1 week post‐V3, participants with Delta first infection had higher inferred LV‐N titres than those with Alpha first infection for LV‐N Ancestral (complete neutralisation vs. 446, *p* = 0.002), LV‐N Alpha (complete neutralisation vs. 624, *p* = 0.01) and LV‐N Delta (complete neutralisation vs. 552, *p* = 0.006). Those with Delta first infection also had considerably higher LV‐Omicron BA.1 titres when compared to individuals with Alpha first infection (1154 vs. 436, *p* = 0.05). No statistical differences were observed for anti‐S (62,861 vs. 33,587 BAU/mL, *p* = 0.31) and LV‐Omicron BA.2 (1232 vs. 770, *p* = 0.36) by variant of first infection (Figure 2). Time since first infection was not significantly associated with the values of titres and did not affect the estimates of the other parameters of the model.

When analysing decay rates, we found no statistical difference by variant of first infection for all antibody responses measured (*p* = 0.27 for anti‐S, larger for nAb*)* (Figure 2). When fitting a model without the interaction term, estimated overall decay rates per week were as follows: anti‐S 6.9% (95% CI 5.8% to 8.3%), LV‐N Ancestral 2.5% (CI 1.2% to 3.7%), LV‐N Alpha 5.5% (CI 4.0% to 6.9%), LV‐N Delta 5.7% (CI 4.3% to 7.1%), LV‐N Omicron BA.1 4.5% (CI 3.3% to 5.6%) and LV‐N Omicron BA.2 3.2% (CI 1.8% to 4.6%).

### 3.2 Comparing Antibody Response at the Time of BA.1 Infection by Variant of First Infection

We compared antibody responses by variant of first infection at the time of BA.1 infections. All 33 participants were included in this analysis. Participants reinfected with BA.1 had their reinfection at a median time of 13 weeks (IQR 11.1–15.3) after V3. We inferred the titres of all participants 12 weeks after V3 and compared them by variant of first infection (Alpha or Delta).

**FIGURE 3:**
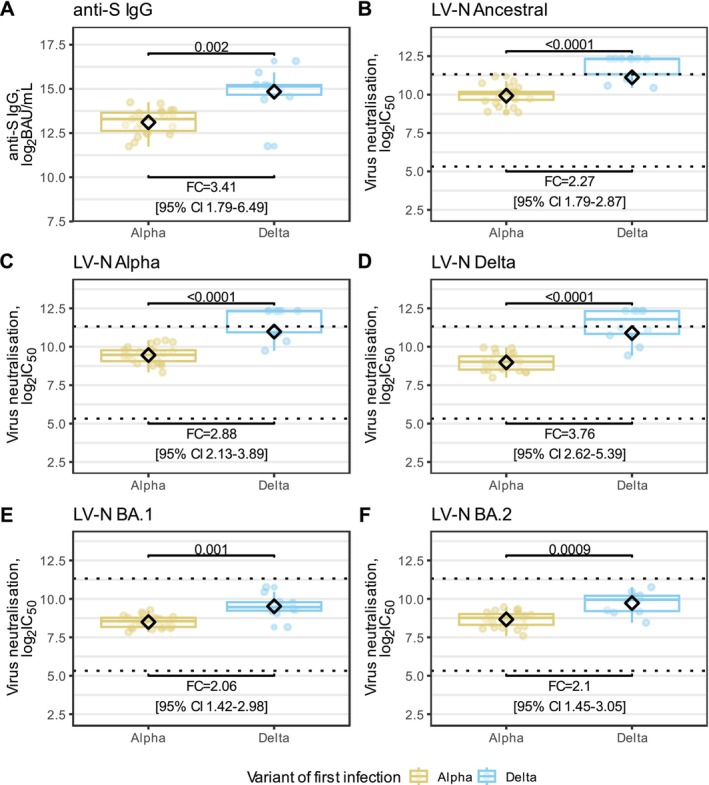
Anti‐S levels and neutralising antibody titres inferred at the time of BA.1 infection, by variant of first infection. (A) Anti‐S IgG modelled at the time of BA.1 infections plotted for cohorts of Alpha or Delta first infection. (B–F) Live virus neutralisation (LV‐N) titres modelled at the time of BA.1 infections for participants plotted for cohorts of Alpha or Delta first infection. Ancestral, Alpha, Delta, Omicron BA.1 and Omicron BA.2 are shown in B to F, respectively. Live virus neutralisation titres are reported as the reciprocal of the dilution at which 50% inhibition of viral entry occurred (IC_50_). In (A–F), all serological data are log2‐transformed and groups were compared. Fold differences between Alpha and Delta first infections are reported on a linear scale. Boxplots shown the interquartile range and medians, with whiskers extending to the lesser of the furthest datapoint, or 1.5× interquartile range. Diamonds indicate the geometric means. In (B–F), dashed horizontal lines indicate the upper and lower bounds of the quantitative range.

Anti‐S levels and nAb titres against all variants were higher in those with Delta first infection compared to Alpha first infection: anti‐S 30458 vs. 8939 BAU/mL (*p* = 0.002), LV‐N Ancestral 2234 vs. 985 (*p* < 0.0001), LV‐N Alpha 2043 vs. 710 (p < 0.0001), LV‐N Delta 1920 vs. 511 (p < 0.0001), LV‐N Omicron BA.1746 vs. 32 (*p* = 0.001) and LV‐N Omicron BA.2861 vs. 409 (*p* = 0.0009) (Figure 3).

### 3.3 Comparing Antibody Response of BA.2‐Infected Participants by First Infection Variant

**FIGURE 4:**
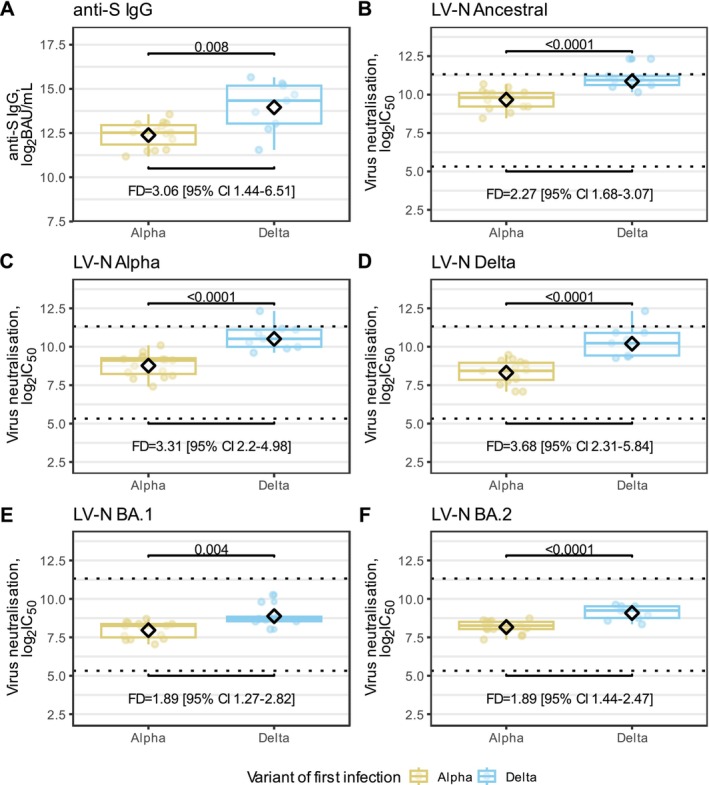
Anti‐S levels and neutralising antibody titres from BA.2 reinfected participants inferred at the time of BA.2 infection, by variant of first infection. (A) anti‐S IgG predicted at the time of BA.2 infections plotted for cohorts of Alpha or Delta first infection. (B–F) Live virus neutralisation (LV‐N) titres predicted at the time of BA.2 infections for participants plotted for cohorts of Alpha or Delta first infection. Ancestral, Alpha, Delta, Omicron BA.1 and Omicron BA.2 are shown in B to F, respectively. Live virus neutralisation titres are reported as the reciprocal of the dilution at which 50% inhibition of viral entry occurred (IC_50_). In (A–F), all serological data are log2‐transformed and groups were compared. Fold differences between Alpha and Delta first infections are reported on a linear scale. Boxplots shown the interquartile range and medians, with whiskers extending to the lesser of the furthest datapoint, or 1.5× interquartile range. Diamonds indicate the geometric means. In (B–F), dashed horizontal lines indicate the upper and lower bounds of the quantitative range.

We compared antibody responses by variant of first infection from individuals reinfected with BA.2, 7 days before their BA.2 infection. Those with Delta first infection had higher inferred anti‐S levels and nAb titres against all variants than compared to individuals with Alpha first infection: anti‐S 16591 vs. 5418 BAU/mL (*p* = 0.01), LV‐N Ancestral 1875 vs. 826 (*p* = 0.0001), LV‐N Alpha 1875 vs. 826 (*p* < 0.0001), LV‐N‐ Delta 1192 vs. 324 (*p* < 0.0001), LV‐N Omicron BA.1477 vs. 252 (*p* = 0.004) and LV‐N Omicron BA.2544 vs. 288 (*p* = 0.001) (Figure 4).

### 3.4 Comparing Neutralisation Responses Among Those With Alpha or Delta First Infection

To assess the potential impact of Alpha and Delta first infection in individuals' neutralisation responses following booster vaccination, we compared the neutralising antibody titres against different variants among those with Alpha (*n* = 23) and Delta (*n* = 10) first infection (separately). We inferred titres at 12 weeks after V3 to all 33 participants.

**FIGURE 5:**
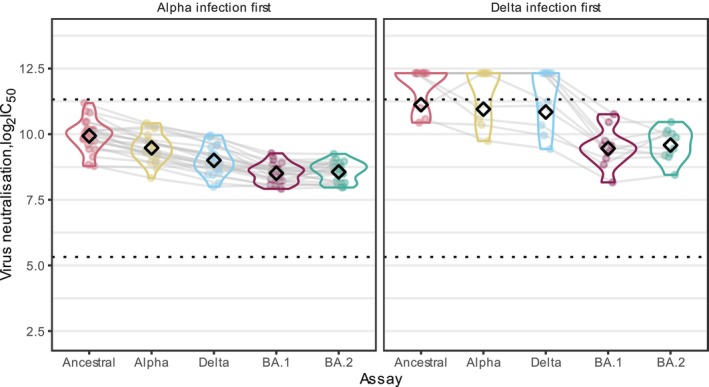
Neutralising antibody titres by variant of first infection at 12 weeks after third vaccine doses. Live virus neutralisation (LV‐N) titres predicted at 12 weeks after third vaccine doses (V3), for participants with Alpha or Delta first infection. Ancestral, Alpha, Delta and Omicron BA.1 and BA.2 are shown. Live virus neutralisation titres are plotted as the log2‐transformed reciprocal of the dilution at which 50% inhibition of viral entry occurred (IC_50_). Individual participants are joined by a grey line and geometric means are shown by black diamonds. Dashed horizontal lines indicate the upper and lower bounds of the quantitative range.

LV‐N Ancestral titres were higher compared to titres against other variants, regardless of variant of first infection. Those with Alpha first infections had higher LV‐N titres against Alpha than against other subsequent variants. For those with Delta first infections, we have still observed higher LV‐N Alpha titres when compared to LV‐N Delta, LV‐N Omicron BA.1 and LV‐N Omicron BA.2 (Figure 5).

## 4 Discussion

Despite a large proportion of the population worldwide being previously exposed to SARS‐CoV‐2 infection and multiple COVID‐19 vaccinations, ensuring protection against SARS‐CoV‐2 emerging variants remains a challenge in the face of ongoing virus evolution. Understanding the impact of first infection variant and how it confers protection to subsequent variants is an important topic given increasingly heterogeneous hybrid immunity. Our study with extensive longitudinal serological data paired with frequent PCR testing is well placed to investigate the impact of first infection variant in protection against Omicron (BA.1 and BA.2) infections.

We found that participants first infected with the Delta variant had higher neutralising antibody titres, immediately after V3, against Ancestral, Alpha and Delta variants than those with Alpha first infection. This trend continued pre‐BA.1 infection and pre‐BA.2 infection. Higher antibody titres for those with Delta compared to Alpha first infection could be related to different immunogenic properties of this variant, which may lead to an enhanced inflammatory response and disease severity [23, 24, 25]. Although our study was not designed to assess disease severity, none of the participants selected for this analysis reported a hospital admission related to their first SARS‐CoV‐2 infection episode.

Our results suggested that a previous Delta infection conferred more protection against Omicron variants than Alpha infection, even when controlling for time since first infection. This is aligned with previous studies, which demonstrated lower protection against Omicron reinfection in those infected before Delta variant emergence when compared to those with previous Delta infection [26, 27]. It has also been demonstrated that, across multiple Omicron waves, those previously infected with more recent variants had increased protection against reinfection compared to earlier variants [28]. These findings are supported by the fact that Delta infection is more effective in inducing high nAb titres against Omicron sub‐lineages as opposed to Alpha, despite BA.1 and BA.2 being antigenically distinct [29].

When comparing an individual's response to different variants separately for Alpha and Delta first infections following V3, higher LV‐N Ancestral titres were observed regardless of the variant of first infection. Although these findings provide no evidence of immunological imprinting by the variant of first infection, they support the immune imprinting effect of their first antigenic exposure (Ancestral SARS‐CoV‐2 virus from vaccination), given most participants (79%) had received at least one vaccine dose before their first infection. Consequently, this demonstrates the immune‐escape potential of early Omicron variants, with reduced cross‐reactivity with predecessors' variants. Other cohorts of individuals with different vaccination and infection records, later infected with Omicron, demonstrated similar patterns of antibody response [18, 30]. This is particularly relevant for vaccine development and supports the strategy of updating SARS‐CoV‐2 booster immunisations with more contemporary antigens to enhance individuals' neutralisation activity against antigenically distinct variants [13].

Our study strengths include the frequent testing and large availability of samples, allowing us to assess the serological response in detail and provide estimates at different timepoints. Our models carefully considered different biases, such as the time of first infection, that could lead to confounded results if not adjusted.

The main limitation in this study is the relatively small sample size, given we only selected participants with Omicron reinfections that had samples available at the time of data cut‐off; therefore, the results should be interpreted with caution. Although this may affect generalisability, our models produced results consistent with those derived from studies using larger sample sizes. In addition, there were not enough participants with distinct vaccination patterns (pre‐ and post‐infection) to support an analysis on its impact on antibody response, which may have influenced our results. Moreover, there are many complexities intrinsic to this analysis, particularly the interconnectedness between time, variant and vaccination. Given the brisk replacement of Alpha by Delta over approximately 2 weeks in 2021, it is challenging to disentangle the effect of time since first infection and variant of first infection. Our models also considered an average time since V3 for all participants, which may have resulted in different calendar times for each participant when calculating pre‐BA.1 infection titres.

In conclusion, our findings demonstrate that an Alpha or Delta primary SARS‐CoV‐2 infection shapes an individual's serological response against a subsequent Omicron infection.

## Author Contributions

A.A., E.J.C., and F.I. were responsible for formal analysis, investigation, methodology, validation, visualisation and writing–original draft. A.A., F.I., E.J.C., A.D.O., S.F., A.C, R.B., G.D., A.H., M.Y.W., S.H, and V.H were responsible for conceptualisation and methodology. S.F, E.J.C., and F.I were responsible for data curation. V.H., S.H., A.C. and R.B. were responsible for supervision. A.A. was responsible for project administration. E.J.C., A.D.O., G.D., A.H., and M.Y.W. were responsible for resources. All authors were responsible for writing–review and editing. All authors had full access to all the data in the study and accept responsibility to submit for publication.

## Funding

This work was supported by the UKHSA, the Medical Research Council (MR/W02067X/1 and MR/X006751/1 to EJC) and the Francis Crick Institute, which receives its core funding from Cancer Research UK (CC2230, CC2087 and CC0102), the UK Medical Research Council (CC2230, CC2087 and CC0102) and the Wellcome Trust (CC2230, CC2087 and CC0102). The funders of the study had no role in study design, data collection, data analysis, data interpretation or writing of the report. The views expressed are those of the authors and not necessarily those of the DHSC, UKHSA, NIHR or The Francis Crick Institute.

## Ethics Statement

The study was approved by the Berkshire Research Ethics Committee, Health Research Authority (IRAS ID 284460, REC reference 20/SC/0230) on 22 May 2020. Participants gave informed consent before joining the study.

## Conflicts of Interest

The authors declare no conflicts of interest.

## Supporting information


**Table S1:** Demographics of the reinfection study grouped by the variant of their first infection.
**Table S2:** Demographics of the re‐infection study grouped by variant of reinfection.
**Figure S1:** Time between first infection, vaccination and Omicron reinfections. Each line represents one participant included in this analysis. Numbers represent first and second infections. Dots represent vaccine doses. For the purpose of this illustration, vaccine doses administrated before first infection are not shown.

## Data Availability

Anonymised data will be made available for secondary analysis to trusted researchers upon reasonable request.

## References

[1] C. Dimeglio , M. Migueres , J. M. Mansuy , et al., “Antibody Titers and Breakthrough Infections With Omicron SARS‐CoV‐2,” Journal of Infection 84, no. 4 (2022): e13–e15, 10.1016/j.jinf.2022.01.04435123960. PMC8812090 35123960

[2] T. Braeye , L. Catteau , R. Brondeel , et al., “Vaccine Effectiveness Against Transmission of Alpha, Delta and Omicron SARS‐CoV‐2‐Infection, Belgian Contact Tracing, 2021–2022,” Vaccine 41, no. 20 (2023): 3292–3300. 37085456 10.1016/j.vaccine.2023.03.069 PMC10073587

[3] UK Health Security Agency SARS‐CoV‐2 variants of concern and variants under investigation in England. Technical briefing 38 2022, https://assets.publishing.service.gov.uk/media/622b4a20e90e070ed8233a05/Technical‐Briefing‐38‐11March2022.pdf.

[4] E. J. M. Monk , S. Foulkes , K. Munro , et al., “Characterisation of the SARS‐CoV‐2 Pandemic in Healthcare Workers Within the United Kingdom: Risk Factors for Infection During Four Successive Waves,” Journal of Infection 90, no. 2 (2024): 106393, https://www.journalofinfection.com/article/S0163‐4453%2824%2900328‐1/fulltext?utm. 39733824 10.1016/j.jinf.2024.106393

[5] V. J. Hall , S. Foulkes , A. Charlett , et al., “SARS‐CoV‐2 Infection Rates of Antibody‐Positive Compared With Antibody‐Negative Health‐Care Workers in England: A Large, Multicentre, Prospective Cohort Study (SIREN),” Lancet 397, no. 10283 (2021): 1459–1469. 33844963 10.1016/S0140-6736(21)00675-9 PMC8040523

[6] A. S. Walker , K. D. Vihta , O. Gethings , et al., “Tracking the Emergence of SARS‐CoV‐2 Alpha Variant in the United Kingdom,” New England Journal of Medicine 385, no. 27 (2021): 2582–2585. 34879193 10.1056/NEJMc2103227 PMC8693687

[7] V. J. Hall , S. Foulkes , A. Saei , et al., “COVID‐19 Vaccine Coverage in Health‐Care Workers in England and Effectiveness of BNT162b2 mRNA Vaccine Against Infection (SIREN): A Prospective, Multicentre, Cohort Study,” Lancet 397, no. 10286 (2021): 1725–1735. 33901423 10.1016/S0140-6736(21)00790-X PMC8064668

[8] C. Trobajo‐Sanmartín , I. Martínez‐Baz , A. Miqueleiz , et al., “Differences in Transmission Between SARS‐CoV‐2 Alpha (B.1.1.7) and Delta (B.1.617.2) Variants,” Microbiology Spectrum 10, no. 2 (2022): e0000822, https://pubmed.ncbi.nlm.nih.gov/35412379/. 35412379 10.1128/spectrum.00008-22 PMC9045255

[9] J. Lopez Bernal , N. Andrews , C. Gower , et al., “Effectiveness of Covid‐19 Vaccines Against the B.1.617.2 (Delta) Variant,” New England Journal of Medicine 385, no. 7 (2021): 585–594. https://www.nejm.org/doi/full/10.1056/NEJMoa2108891. 34289274 10.1056/NEJMoa2108891 PMC8314739

[10] Y. Goldberg , M. Mandel , Y. M. Bar‐On , et al., “Protection and Waning of Natural and Hybrid Immunity to SARS‐CoV‐2,” New England Journal of Medicine 386, no. 23 (2022): 2201–2212. 35613036 10.1056/NEJMoa2118946 PMC9165562

[11] N. Lasrado and D. H. Barouch , “SARS‐CoV‐2 Hybrid Immunity: The Best of Both Worlds,” Journal of Infectious Diseases 228, no. 10 (2023): 1311–1313. 37592872 10.1093/infdis/jiad353

[12] D. Y. Lin , Y. Xu , Y. Gu , D. Zeng , S. K. Sunny , and Z. Moore , “Durability of Bivalent Boosters Against Omicron Subvariants,” New England Journal of Medicine 388, no. 19 (2023): 1818–1820. 37043647 10.1056/NEJMc2302462 PMC10120009

[13] M. Koutsakos and A. H. Ellebedy , “Immunological Imprinting: Understanding COVID‐19,” Immunity 56, no. 5 (2023): 909–913. 37105169 10.1016/j.immuni.2023.04.012 PMC10113596

[14] J. Torresi and M. A. Edeling , “Immune Imprinting of SARS‐CoV‐2 Responses: Changing First Immune Impressions,” mSphere 9, no. 4 (2024): e0075823. 38477577 10.1128/msphere.00758-23 PMC11036796

[15] K. Khan , F. Karim , S. Cele , et al., “Omicron Infection Enhances Delta Antibody Immunity in Vaccinated Persons,” Nature 607 (2022): 356–359. 35523247 10.1038/s41586-022-04830-x PMC9279144

[16] M. A. Tortorici , A. Addetia , A. J. Seo , et al., “Persistent Immune Imprinting Occurs After Vaccination With the COVID‐19 XBB.1.5 mRNA Booster in Humans,” Immunity 57, no. 4 (2024): 904–911.e4. 38490197 10.1016/j.immuni.2024.02.016 PMC12360627

[17] A. Yisimayi , W. Song , J. Wang , et al., “Repeated Omicron Exposures Override Ancestral SARS‐CoV‐2 Immune Imprinting,” Nature [Internet] 625, no. 7993 (2024): 148–156. 10.1038/s41586-023-06753-7 PMC10764275 37993710

[18] A. K. Wheatley , A. Fox , H. X. Tan , et al., “Immune Imprinting and SARS‐CoV‐2 Vaccine Design,” Trends in Immunology 42, no. 11 (2021): 956–959. 34580004 10.1016/j.it.2021.09.001 PMC8440232

[19] A. Rössler , L. Knabl , D. von Laer , and J. Kimpel , “Neutralization Profile After Recovery From SARS‐CoV‐2 Omicron Infection,” New England Journal of Medicine 386, no. 18 (2022): 1764–1766. 35320661 10.1056/NEJMc2201607 PMC9006769

[20] S. E. Wallace , V. Hall , A. Charlett , et al., “Impact of Prior SARS‐CoV‐2 Infection and COVID‐19 Vaccination on the Subsequent Incidence of COVID‐19: A Multicentre Prospective Cohort Study Among UK Healthcare Workers – The SIREN (Sarscov2 Immunity & REinfection EvaluatioN) Study Protocol,” BMJ Open 12 (2022): e054336. 10.1136/bmjopen-2021-054336 PMC9240450 35768083

[21] M. Wu , E. C. Wall , E. J. Carr , et al., “Three‐Dose Vaccination Elicits Neutralising Antibodies Against Omicron,” Lancet 399, no. 10326 (2022): 715–717. 35065005 10.1016/S0140-6736(22)00092-7 PMC8769665

[22] A. Atti , F. Insalata , E. J. Carr , et al., “Antibody Correlates of Protection Against Delta Infection After Vaccination: A Nested Case‐Control Within the UK‐Based SIREN Study,” Journal of infection 87, no. 5 (2023): 420–427. 37689394 10.1016/j.jinf.2023.07.007

[23] K. A. Twohig , T. Nyberg , A. Zaidi , et al., “Hospital Admission and Emergency Care Attendance Risk for SARS‐CoV‐2 Delta (B.1.617.2) Compared With Alpha (B.1.1.7) Variants of Concern: A Cohort Study,” Lancet Infectious Diseases 22, no. 1 (2021): 35–42. 34461056 10.1016/S1473-3099(21)00475-8 PMC8397301

[24] C. H. Luo , C. P. Morris , J. Sachithanandham , et al., “Infection With the SARS‐CoV‐2 Delta Variant Is Associated With Higher Recovery of Infectious Virus Compared to the Alpha Variant in Both Unvaccinated and Vaccinated Individuals,” Clinical Infectious Diseases 75, no. 1 (2021): e715–e725. 10.1093/cid/ciab986 PMC8903351 34922338

[25] K. S. Lee , T. Y. Wong , B. P. Russ , et al., “SARS‐CoV‐2 Delta Variant Induces Enhanced Pathology and Inflammatory Responses in K18‐hACE2 Mice,” PLoS One 17, no. 8 (2022): e0273430. 36037222 10.1371/journal.pone.0273430 PMC9423646

[26] A. A. Powell , F. Kirsebom , J. Stowe , et al., “Protection Against Symptomatic Infection with Delta (B.1.617.2) and Omicron (B.1.1.529) BA.1 and BA.2 SARS‐CoV‐2 Variants After Previous Infection and Vaccination in Adolescents in England, August, 2021–March, 2022: A National, Observational, Test‐negative, Case‐control Study,” Lancet Infectious Diseases 23, no. 4 (2022): 435–444. 36436536 10.1016/S1473-3099(22)00729-0 PMC10032664

[27] E. T. Chin , D. Leidner , L. Lamson , et al., “Protection Against Omicron From Vaccination and Previous Infection in a Prison System,” New England Journal of Medicine 387, no. 19 (2022): 1770–1782. 36286260 10.1056/NEJMoa2207082 PMC9634863

[28] J. Wei , N. Stoesser , P. C. Matthews , et al., “Risk of SARS‐CoV‐2 Reinfection During Multiple Omicron Variant Waves in the UK General Population,” Nature Communications 15, no. 1 (2024): 1008. 10.1038/s41467-024-44973-1 PMC10837445 38307854

[29] A. Rössler , A. Netzl , L. Knabl , et al., “BA.2 and BA.5 Omicron Differ Immunologically From Both BA.1 Omicron and Pre‐Omicron Variants,” Nature Communications 13, no. 1 (2022): 7701. 10.1038/s41467-022-35312-3 PMC9745279 36513653

[30] C. J. Reynolds , C. Pade , J. M. Gibbons , et al., “Immune Boosting by B.1.1.529 (Omicron) Depends on Previous SARS‐CoV‐2 Exposure,” Science 377, no. 6603 (2022): eabq1841. 35699621 10.1126/science.abq1841 PMC9210451

