# Supplementary material for: Impact of First SARS‐CoV‐2 Infection Variant on Serological Responses Against Omicron: Findings From the SIREN Study
Source: Influenza Other Respir Viruses. 2026 Feb 22;20(2):e70204. doi: 10.1111/irv.70204 (PMC12928044; doi:10.1111/irv.70204)
Supplement: Supplementary file 1 — Table S1: Demographics of the reinfection study grouped by the variant of their first infection. Table S2: Demographics of the re‐infection study grouped by variant of reinfection. Figure S1: Time between first infection, vaccination and Omicron reinfections. Each line represents one participant included in this analysis. Numbers represent first and second infections. Dots represent vaccine doses. For the purpose of this illustration, vaccine doses administrated before first infection are not shown. [file IRV-20-e70204-s001.docx]

**SUPPLEMENTARY MATERIAL**

**Supplementary Tables and Figures**

| **Characteristic** | | **Alpha**, N = 23*^1^* | | **Delta**, N = 10*^1^* | |
| --- | --- | --- | --- | --- | --- |
| Sex | |  | |  | |
| Female | | 18 (78%) | | 9 (90%) | |
| Male | | 5 (22%) | | 1 (10%) | |
| Age | |  | |  | |
| Under 25 | | 1 (4.3%) | | 0 (0%) | |
| 25 to 34 | | 7 (30%) | | 2 (20%) | |
| 35 to 44 | | 4 (17%) | | 3 (30%) | |
| 45 to 54 | | 10 (43%) | | 2 (20%) | |
| Over 55 | | 1 (4.3%) | | 3 (30%) | |
| Ethnic group | |  | |  | |
| White | | 20 (87%) | | 9 (90%) | |
| Asian | | 3 (13%) | | 0 (0%) | |
| Black | | 0 (0%) | | 1 (10%) | |
| Patient facing | |  | |  | |
| Yes | | 20 (87%) | | 7 (70%) | |
| No | | 3 (13%) | | 3 (30%) | |
| Comorbidities | |  | |  | |
| No medical condition | | 14 (61%) | | 8 (80%) | |
| Chronic respiratory conditions | | 6 (26%) | | 2 (20%) | |
| Chronic non respiratory conditions | | 3 (13%) | | 0 (0%) | |
| *^1^* n (%) | | | | | |

**Supplementary Table 1. Demographics of the reinfection study grouped by the variant of their first infection.**

| **Characteristic** | | **BA.1**, N = 9*^1^* | | **BA.2**, N = 24*^1^* | |
| --- | --- | --- | --- | --- | --- |
| Sex | |  | |  | |
| Female | | 6 (67%) | | 21 (88%) | |
| Male | | 3 (33%) | | 3 (13%) | |
| Age | |  | |  | |
| Under 25 | | 1 (11%) | | 0 (0%) | |
| 25 to 34 | | 1 (11%) | | 8 (33%) | |
| 35 to 44 | | 4 (44%) | | 3 (13%) | |
| 45 to 54 | | 3 (33%) | | 9 (38%) | |
| Over 55 | | 0 (0%) | | 4 (17%) | |
| Ethnic group | |  | |  | |
| White | | 8 (89%) | | 21 (88%) | |
| Asian | | 1 (11%) | | 2 (8.3%) | |
| Black | | 0 (0%) | | 1 (4.2%) | |
| Patient facing | |  | |  | |
| Yes | | 9 (100%) | | 18 (75%) | |
| No | | 0 (0%) | | 6 (25%) | |
| Comorbidities | |  | |  | |
| No medical condition | | 8 (89%) | | 14 (58%) | |
| Chronic respiratory conditions | | 0 (0%) | | 8 (33%) | |
| Chronic non respiratory conditions | | 1 (11%) | | 2 (8.3%) | |
| *^1^* n (%) | | | | | |

**Supplementary Table 2. Demographics of the re-infection study grouped by variant of reinfection.**

**
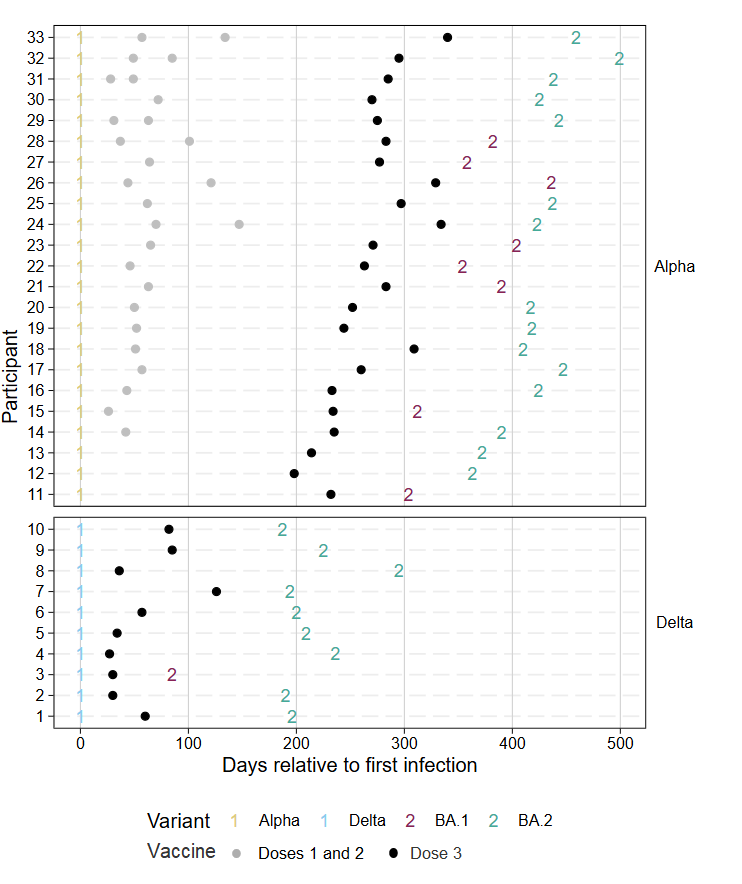
**

**Supplementary Figure 1. Time between first infection, vaccination and Omicron reinfections.** Each line represents one participant included in this analysis. Numbers represent first and second infections. Dots represent vaccine doses. For the purpose of this illustration, vaccine doses administrated before first infection are not shown.

**SIREN Study Group members**

| **Site Name** | | **First Name** | **Surname** | |
| --- | --- | --- | --- | --- |
| ALDER HEY CHILDREN'S NHS FOUNDATION TRUST | | Stephen | McWilliam | |
| ALDER HEY CHILDREN'S NHS FOUNDATION TRUST | | Beatriz | Larru | |
| ANEURIN BEVAN UNIVERSITY LHB | | John | Northfield | |
| ANEURIN BEVAN UNIVERSITY LHB | | Sean | Cutler | |
| ASHFORD AND ST PETER'S HOSPITALS NHS FOUNDATION TRUST | | Stephen | Winchester | |
| ASHFORD AND ST PETER'S HOSPITALS NHS FOUNDATION TRUST | | Samuel | Rowley | |
| BASILDON AND THURROCK UNIVERSITY HOSPITALS NHS FOUNDATION TRUST | | Anirudda | Pai | |
| BASILDON AND THURROCK UNIVERSITY HOSPITALS NHS FOUNDATION TRUST | | Stacey | Pepper | |
| BEDFORDSHIRE HOSPITALS NHS FOUNDATION TRUST | | Simantee | Guha | |
| BEDFORDSHIRE HOSPITALS NHS FOUNDATION TRUST | | Philippa | Bakker | |
| BELFAST HEALTH & SOCIAL CARE TRUST | | Clodagh | Loughrey | |
| BETSI CADWALADR UNIVERSITY LHB | | Christian | Subbe | |
| BETSI CADWALADR UNIVERSITY LHB | | Caroline | Mulvaney Jones | |
| BIRMINGHAM AND SOLIHULL MENTAL HEALTH NHS FOUNDATION TRUST | | Manny | Bagary | |
| BIRMINGHAM AND SOLIHULL MENTAL HEALTH NHS FOUNDATION TRUST | | Nadezda | Starkova | |
| BLACK COUNTRY HEALTHCARE NHS FOUNDATION TRUST | | Alison | Grant | |
| BLACK COUNTRY HEALTHCARE NHS FOUNDATION TRUST | | Rebecca | Temple-Purcell | |
| BLACKPOOL TEACHING HOSPITALS NHS FOUNDATION TRUST | | Joanne | Howard | |
| BLACKPOOL TEACHING HOSPITALS NHS FOUNDATION TRUST | | Emma | Ward | |
| BOLTON NHS FOUNDATION TRUST | | Chinari | Subudhi | |
| BOLTON NHS FOUNDATION TRUST | | Scott | Latham | |
| BRIGHTON AND SUSSEX UNIVERSITY HOSPITALS NHS TRUST | | Bethany | Davies | |
| BRIGHTON AND SUSSEX UNIVERSITY HOSPITALS NHS TRUST | | Marion | Campbell | |
| BUCKINGHAMSHIRE HEALTHCARE NHS TRUST | | Nick | Wong | |
| BUCKINGHAMSHIRE HEALTHCARE NHS TRUST | | Ruth | Penn | |
| CALDERDALE AND HUDDERSFIELD NHS FOUNDATION TRUST | | N | Wong | |
| CALDERDALE AND HUDDERSFIELD NHS FOUNDATION TRUST | | Gavin | Boyd | |
| CENTRAL AND NORTH WEST LONDON NHS FOUNDATION TRUST | | Abigail | Severn | |
| CENTRAL AND NORTH WEST LONDON NHS FOUNDATION TRUST | | Alejandro | Arenas-Pinto | |
| CHESTERFIELD ROYAL HOSPITAL NHS FOUNDATION TRUST | | Thomas | Spencer | |
| CHESTERFIELD ROYAL HOSPITAL NHS FOUNDATION TRUST | | Edward | Harris | |
| CORNWALL PARTNERSHIP NHS FOUNDATION TRUST | | Susan | Greenwood | |
| CORNWALL PARTNERSHIP NHS FOUNDATION TRUST | | Angela | Pengilly | |
| COUNTESS OF CHESTER HOSPITAL NHS FOUNDATION TRUST | | Kim | Wells | |
| COUNTESS OF CHESTER HOSPITAL NHS FOUNDATION TRUST | | Therea | Barnes | |
| CROYDON HEALTH SERVICES NHS TRUST | | C | Jones | |
| CROYDON HEALTH SERVICES NHS TRUST | | Banerjee | Subhro-Osuji | |
| CWM TAF MORGANNWG UNIVERSITY LHB | | John | Geen | |
| CWM TAF MORGANNWG UNIVERSITY LHB | | Carla | Pothecary | |
| DARTFORD AND GRAVESHAM NHS TRUST | | Tracy | Edmunds | |
| DARTFORD AND GRAVESHAM NHS TRUST | | Nihil | Chitalia | |
| DERBYSHIRE COMMUNITY HEALTH SERVICES NHS FOUNDATION TRUST | | Ben | Pearson | |
| DERBYSHIRE COMMUNITY HEALTH SERVICES NHS FOUNDATION TRUST | | Sarah | Creer | |
| DEVON PARTNERSHIP NHS TRUST | | Clare | McAdam | |
| DEVON PARTNERSHIP NHS TRUST | | Natalie | Crooks | |
| DONCASTER AND BASSETLAW TEACHING HOSPITALS NHS FOUNDATION TRUST | | Anna | Grice | |
| DONCASTER AND BASSETLAW TEACHING HOSPITALS NHS FOUNDATION TRUST | | Ken | Agwuh | |
| DORSET COUNTY HOSPITAL NHS FOUNDATION TRUST | | Jennifer | Graves | |
| DORSET HEALTHCARE UNIVERSITY NHS FOUNDATION TRUST | | Paul | Walters | |
| EAST SUFFOLK AND NORTH ESSEX NHS FOUNDATION TRUST | | Luke | Bedford | |
| EAST SUFFOLK AND NORTH ESSEX NHS FOUNDATION TRUST | | Paul | Ridley | |
| EAST SUSSEX HEALTHCARE NHS TRUST | | Anna | Cowley | |
| EAST SUSSEX HEALTHCARE NHS TRUST | | Janet | Sinclair | |
| EPSOM AND ST HELIER UNIVERSITY HOSPITALS NHS TRUST | | Helen | Johnstone | |
| EPSOM AND ST HELIER UNIVERSITY HOSPITALS NHS TRUST | | Neringa | Vilimiene | |
| FRIMLEY HEALTH NHS FOUNDATION TRUST (Frimley Park hospital) | | Manjula | Meda | |
| FRIMLEY HEALTH NHS FOUNDATION TRUST (Wexham Park hospital) | | Nicky | Barnes | |
| GEORGE ELIOT HOSPITAL NHS TRUST | | Simon | Brake | |
| GEORGE ELIOT HOSPITAL NHS TRUST | | David | Boss | |
| GLOUCESTERSHIRE HOSPITALS NHS FOUNDATION TRUST | | Chris | Ford | |
| GLOUCESTERSHIRE HOSPITALS NHS FOUNDATION TRUST | | Amanda | Selassie | |
| GOLDEN JUBILEE NATIONAL HOSPITAL | | Catherine | Sinclair | |
| GOLDEN JUBILEE NATIONAL HOSPITAL | | Val | Irvine | |
| GREAT WESTERN HOSPITALS NHS FOUNDATION TRUST | | Badrinathan | Chandrasekaran | |
| GREAT WESTERN HOSPITALS NHS FOUNDATION TRUST | | Eva | Fraile | |
| HAMPSHIRE HOSPITALS NHS FOUNDATION TRUST | | Claire | Thomas | |
| HAMPSHIRE HOSPITALS NHS FOUNDATION TRUST | | Ina | Hoad | |
| HOUNSLOW AND RICHMOND COMMUNITY HEALTHCARE NHS TRUST | | John | Omany | |
| HOUNSLOW AND RICHMOND COMMUNITY HEALTHCARE NHS TRUST | | Shekoo | Mackay | |
| HULL UNIVERSITY TEACHING HOSPITALS NHS TRUST | | Phillipa | Burns | |
| HULL UNIVERSITY TEACHING HOSPITALS NHS TRUST | | Nicholas | Easom | |
| HYWEL DDA UNIVERSITY LHB | | Tracy | Lewis | |
| IMPERIAL COLLEGE HEALTHCARE NHS TRUST | | Frances | Frances | |
| IMPERIAL COLLEGE HEALTHCARE NHS TRUST | | Graham | Pickard | |
| ISLE OF WIGHT NHS TRUST | | Emily | Macnaughton | |
| ISLE OF WIGHT NHS TRUST | | Sarah | Knight | |
| JAMES PAGET UNIVERSITY HOSPITALS NHS FOUNDATION TRUST | | Davis | Nwaka | |
| JAMES PAGET UNIVERSITY HOSPITALS NHS FOUNDATION TRUST | | Christian | Hacon | |
| KING'S COLLEGE HOSPITAL NHS FOUNDATION TRUST | | Jasmin | Islam | |
| KING'S COLLEGE HOSPITAL NHS FOUNDATION TRUST | | Ray | Chaudhuri | |
| LANCASHIRE & SOUTH CUMBRIA NHS FOUNDATION TRUST | | Robert | Shorten | |
| LANCASHIRE & SOUTH CUMBRIA NHS FOUNDATION TRUST | | Kathryn | Hollinshead | |
| LEEDS TEACHING HOSPITALS NHS TRUST | | Jacqueline | Brandon | |
| LEEDS TEACHING HOSPITALS NHS TRUST | | Kyra | Holliday | |
| LEICESTERSHIRE PARTNERSHIP NHS TRUST | | Sarah | Baillon | |
| LEICESTERSHIRE PARTNERSHIP NHS TRUST | | Samantha | Hamer | |
| LEWISHAM AND GREENWICH NHS TRUST | | Judith | Russell | |
| LEWISHAM AND GREENWICH NHS TRUST | | A | Shah | |
| LINCOLNSHIRE PARTNERSHIP NHS FOUNDATION TRUST | | Kelly | Moran | |
| LINCOLNSHIRE PARTNERSHIP NHS FOUNDATION TRUST | | Vijayendra | Waykar | |
| LIVERPOOL UNIVERSITY HOSPITALS NHS FOUNDATION TRUST | | Anu | Chawla | |
| LIVERPOOL UNIVERSITY HOSPITALS NHS FOUNDATION TRUST | | Fran | Westwell | |
| LONDON NORTH WEST UNIVERSITY HEALTHCARE NHS TRUST | | P. | Papinni | |
| LONDON NORTH WEST UNIVERSITY HEALTHCARE NHS TRUST | | Ekaterina | Watson | |
| MAIDSTONE AND TUNBRIDGE WELLS NHS TRUST | | Claire | Pegg | |
| MAIDSTONE AND TUNBRIDGE WELLS NHS TRUST | | Maureen | Williams | |
| MANCHESTER UNIVERSITY NHS FOUNDATION TRUST | | Alexander | Horsley | |
| MANCHESTER UNIVERSITY NHS FOUNDATION TRUST | | Shazaad | Ahmad | |
| MID CHESHIRE HOSPITALS NHS FOUNDATION TRUST | | Elijah | Matovu | |
| MID CHESHIRE HOSPITALS NHS FOUNDATION TRUST | | Claire | Gabriel | |
| MID ESSEX HOSPITAL SERVICES NHS TRUST | | Lauren | Sach | |
| MID ESSEX HOSPITAL SERVICES NHS TRUST | | Yvonne | Lester | |
| MID YORKSHIRE HOSPITALS NHS TRUST | | AJ. | Ashcroft | |
| MID YORKSHIRE HOSPITALS NHS TRUST | | Ismaelette | Del Rosario | |
| MOORFIELDS EYE HOSPITAL NHS FOUNDATION TRUST | | Roxanne | Crosby-Nwaobi | |
| MOORFIELDS EYE HOSPITAL NHS FOUNDATION TRUST | | Chloe | Reeks | |
| NHS BORDERS | | Joy | Dawson | |
| NHS BORDERS | | Lauren | Finlayson | |
| NHS FIFE | | Devesh | Dhasmana | |
| NHS FIFE | | Susan | Fowler | |
| NHS FORTH VALLEY | | Euan | Cameron | |
| NHS FORTH VALLEY | | Anne | Todd | |
| NHS GRAMPIAN | | Vhair | Bateman | |
| NHS GRAMPIAN | | Sally | Mavin | |
| NHS GREATER GLASGOW AND CLYDE | | Antonia | Ho | |
| NHS GREATER GLASGOW AND CLYDE | | Michael | Murphy | |
| NHS HIGHLAND | | Andrew | Gibson | |
| NHS HIGHLAND | | Alexandra | Cochrane | |
| NHS LANARKSHIRE | | Manish | Patel | |
| NHS LANARKSHIRE | | Berni | Welsh | |
| NHS LOTHIAN | | Kate | Templeton | |
| NHS LOTHIAN | | Sam | Donaldson | |
| NHS WESTERN ISLES | | Martin | Malcolm | |
| NHS WESTERN ISLES | | Beth | Smith | |
| NORFOLK AND NORWICH UNIVERSITY HOSPITALS NHS FOUNDATION TRUST | | Ngozi | Elumogo | |
| NORFOLK AND NORWICH UNIVERSITY HOSPITALS NHS FOUNDATION TRUST | | Louise | Coke | |
| NORTH CUMBRIA INTEGRATED CARE NHS FOUNDATION TRUST | | Edward | Barton | |
| NORTH CUMBRIA INTEGRATED CARE NHS FOUNDATION TRUST | | Beverley | Wilkinson | |
| NORTH MIDDLESEX UNIVERSITY HOSPITAL NHS TRUST | | Mariyam | Mirfenderesky | |
| NORTH MIDDLESEX UNIVERSITY HOSPITAL NHS TRUST | | Swati | Jain | |
| NORTH WEST ANGLIA NHS FOUNDATION TRUST | | Kanchan | Rege | |
| NORTH WEST ANGLIA NHS FOUNDATION TRUST | | Janki | Bhayani | |
| NORTHERN DEVON HEALTHCARE NHS TRUST | | Tom | Lewis | |
| NORTHERN DEVON HEALTHCARE NHS TRUST | | M | Howard | |
| NORTHERN HEALTH & SOCIAL CARE TRUST | | Elinor | Hanna | |
| NORTHERN HEALTH & SOCIAL CARE TRUST | | Frances | Johnston | |
| NORTHERN LINCOLNSHIRE AND GOOLE NHS FOUNDATION TRUST | | Jonathan | Hatton | |
| NORTHERN LINCOLNSHIRE AND GOOLE NHS FOUNDATION TRUST | | Peter | Cowling | |
| NOTTINGHAM UNIVERSITY HOSPITALS NHS TRUST | | Sarah | Brand | |
| NOTTINGHAM UNIVERSITY HOSPITALS NHS TRUST | | Jack | Squires | |
| POOLE HOSPITAL NHS FOUNDATION TRUST | | Liz | Sheridan | |
| POOLE HOSPITAL NHS FOUNDATION TRUST | | Charlotte | Barclay | |
| PORTSMOUTH HOSPITALS NHS TRUST | | Johanna | Mouland | |
| PORTSMOUTH HOSPITALS NHS TRUST | | Karen | Hudson | |
| POWYS TEACHING LHB | | Jayne | Goodwin | |
| POWYS TEACHING LHB | | Chris | Norman | |
| QUEEN VICTORIA HOSPITAL NHS FOUNDATION TRUST | | Julian | Giles | |
| ROYAL BERKSHIRE NHS FOUNDATION TRUST | | Tim | Parke | |
| ROYAL BERKSHIRE NHS FOUNDATION TRUST | | Maya | Joseph | |
| ROYAL CORNWALL HOSPITALS NHS TRUST | | Duncan | Browne | |
| ROYAL CORNWALL HOSPITALS NHS TRUST | | H | Chenoweth | |
| ROYAL DEVON AND EXETER NHS FOUNDATION TRUST | | Cressida | Auckland | |
| ROYAL DEVON AND EXETER NHS FOUNDATION TRUST | | Stephanie | Prince | |
| ROYAL FREE LONDON NHS FOUNDATION TRUST | | Alison | Rodger | |
| ROYAL FREE LONDON NHS FOUNDATION TRUST | | Tabitha | Mahungu | |
| ROYAL NATIONAL ORTHOPAEDIC HOSPITAL NHS TRUST | | Simon | Warren | |
| ROYAL NATIONAL ORTHOPAEDIC HOSPITAL NHS TRUST | | Esther | Hanison | |
| ROYAL PAPWORTH HOSPITAL NHS FOUNDATION TRUST | | Sumita | Pai | |
| ROYAL PAPWORTH HOSPITAL NHS FOUNDATION TRUST | | Allison | Doel | |
| ROYAL SURREY COUNTY HOSPITAL NHS FOUNDATION TRUST | | Chery | Marriott | |
| ROYAL SURREY COUNTY HOSPITAL NHS FOUNDATION TRUST | | Charles | Piercy | |
| ROYAL UNITED HOSPITALS BATH NHS FOUNDATION TRUST | | Debbie | Delgado | |
| ROYAL UNITED HOSPITALS BATH NHS FOUNDATION TRUST | | Julia | Vasant | |
| ROYAL UNITED HOSPITALS BATH NHS FOUNDATION TRUST | | Deborah | Howcroft | |
| ROYAL UNITED HOSPITALS BATH NHS FOUNDATION TRUST | | Sarah | Meisner | |
| SALISBURY NHS FOUNDATION TRUST | | Abby | Rand | |
| SALISBURY NHS FOUNDATION TRUST | | Catherine | Thompson | |
| SALISBURY NHS FOUNDATION TRUST | | Holly | Morgan | |
| SANDWELL AND WEST BIRMINGHAM HOSPITALS NHS TRUST | | Ash | Turner | |
| SANDWELL AND WEST BIRMINGHAM HOSPITALS NHS TRUST | | Anne | Hayes | |
| SHEFFIELD CHILDREN'S NHS FOUNDATION TRUST | | Fiona | Shackley | |
| SHEFFIELD CHILDREN'S NHS FOUNDATION TRUST | | James | Pethick | |
| SHEFFIELD TEACHING HOSPITALS NHS FOUNDATION TRUST | | Thushan | de Silva | |
| SHEFFIELD TEACHING HOSPITALS NHS FOUNDATION TRUST | | Helen | Shulver | |
| SHERWOOD FOREST HOSPITALS NHS FOUNDATION TRUST | | Lynne | Allsop | |
| SHERWOOD FOREST HOSPITALS NHS FOUNDATION TRUST | | Shrikant | Ambalkar | |
| SHREWSBURY AND TELFORD HOSPITAL NHS TRUST | | Mandy | Carnahan | |
| SHREWSBURY AND TELFORD HOSPITAL NHS TRUST | | Mandy | Beekes | |
| SHROPSHIRE COMMUNITY HEALTH NHS TRUST | | Johanne | Tomlinson | |
| SOLENT NHS TRUST | | Cathy | Price | |
| SOMERSET NHS FOUNDATION TRUST | | Justin | Pepperell | |
| SOMERSET NHS FOUNDATION TRUST | | Kate | James | |
| SOUTH EASTERN HEALTH & SOCIAL CARE | | Yuri | Protaschik | |
| SOUTH EASTERN HEALTH & SOCIAL CARE | | Susan | Regan | |
| SOUTHEND UNIVERSITY HOSPITAL NHS FOUNDATION TRUST | | John | Day | |
| SOUTHEND UNIVERSITY HOSPITAL NHS FOUNDATION TRUST | | Swapna | Kunhunny | |
| SOUTHERN HEALTH & SOCIAL CARE TRUST | | Angel | Boulos | |
| SOUTHERN HEALTH & SOCIAL CARE TRUST | | Fiona | Thompson | |
| SOUTHPORT AND ORMSKIRK HOSPITAL NHS TRUST | | Katherine | Gray | |
| SOUTHPORT AND ORMSKIRK HOSPITAL NHS TRUST | | Kerryanne | Brown | |
| ST GEORGE'S UNIVERSITY HOSPITALS NHS FOUNDATION TRUST | | Tim | Planche | |
| ST GEORGE'S UNIVERSITY HOSPITALS NHS FOUNDATION TRUST | | Angela | Houston | |
| ST HELENS AND KNOWSLEY TEACHING HOSPITALS NHS TRUST | | Rowan | Pritchard-Jones | |
| ST HELENS AND KNOWSLEY TEACHING HOSPITALS NHS TRUST | | Diane | Wycherley | |
| STOCKPORT NHS FOUNDATION TRUST | | Sharman | Harris | |
| STOCKPORT NHS FOUNDATION TRUST | | Barzo | Faris | |
| SURREY AND SUSSEX HEALTHCARE NHS TRUST | | Kofi | Nimako | |
| SURREY AND SUSSEX HEALTHCARE NHS TRUST | | Simon | Bax | |
| SWANSEA BAY UNIVERSITY LHB | | Rebeccah | Thomas | |
| SWANSEA BAY UNIVERSITY LHB | | Steve | Bain | |
| THE CLATTERBRIDGE CANCER CENTRE NHS FOUNDATION TRUST | | Sheena | Khanduri | |
| THE CLATTERBRIDGE CANCER CENTRE NHS FOUNDATION TRUST | | Nagesh | Kalakonda | |
| THE DUDLEY GROUP NHS FOUNDATION TRUST | | Helen | Ashby | |
| THE HILLINGDON HOSPITALS NHS FOUNDATION TRUST | | Ayida | Gubby | |
| THE HILLINGDON HOSPITALS NHS FOUNDATION TRUST | | Natasha | Mahabir | |
| THE NEWCASTLE UPON TYNE HOSPITALS NHS FOUNDATION TRUST | | Brendan | Payne | |
| THE NEWCASTLE UPON TYNE HOSPITALS NHS FOUNDATION TRUST | | Jayne | Harwood | |
| THE PRINCESS ALEXANDRA HOSPITAL NHS TRUST | | Kathryn | Court | |
| THE PRINCESS ALEXANDRA HOSPITAL NHS TRUST | | Nikki | White | |
| THE ROBERT JONES AND AGNES HUNT ORTHOPAEDIC HOSPITAL NHS FOUNDATION TRUST | | Ruth | Longfellow | |
| THE ROYAL BOURNEMOUTH AND CHRISTCHURCH HOSPITALS NHS FOUNDATION TRUST | | Mihye | Lee | |
| THE ROYAL WOLVERHAMPTON NHS TRUST | | Clare | Ford | |
| THE ROYAL WOLVERHAMPTON NHS TRUST | | Marie | Green | |
| TORBAY AND SOUTH DEVON NHS FOUNDATION TRUST | | Kelly | Barrett | |
| TORBAY AND SOUTH DEVON NHS FOUNDATION TRUST | | Matthew | Halkes | |
| UNITED LINCOLNSHIRE HOSPITALS NHS TRUST | | Alun | Roebuck | |
| UNIVERSITY HOSPITAL SOUTHAMPTON NHS FOUNDATION TRUST | | Eleri | Wilson-Davies | |
| UNIVERSITY HOSPITALS BRISTOL AND WESTON NHS FOUNDATION TRUST | | Rajeka | Lazarus | |
| UNIVERSITY HOSPITALS BRISTOL AND WESTON NHS FOUNDATION TRUST | | Aaran | Sinclair | |
| UNIVERSITY HOSPITALS COVENTRY AND WARWICKSHIRE NHS TRUST | | N | Aldridge | |
| UNIVERSITY HOSPITALS COVENTRY AND WARWICKSHIRE NHS TRUST | | Lisa | Berry | |
| UNIVERSITY HOSPITALS OF DERBY AND BURTON NHS FOUNDATION TRUST | | L | Berry | |
| UNIVERSITY HOSPITALS OF DERBY AND BURTON NHS FOUNDATION TRUST | | Frances | Game | |
| UNIVERSITY HOSPITALS OF LEICESTER NHS TRUST | | Christopher | Holmes | |
| UNIVERSITY HOSPITALS OF LEICESTER NHS TRUST | | Martin | Wiselka | |
| UNIVERSITY HOSPITALS OF MORECAMBE BAY NHS FOUNDATION TRUST | | Timothy | Gatheral | |
| UNIVERSITY HOSPITALS OF MORECAMBE BAY NHS FOUNDATION TRUST | | Lynda | Fothergill | |
| UNIVERSITY HOSPITALS PLYMOUTH NHS TRUST | | David | Hilton | |
| UNIVERSITY HOSPITALS PLYMOUTH NHS TRUST | | Hannah | Jory | |
| VELINDRE NHS TRUST | | Charlotte | Young | |
| VELINDRE NHS TRUST | | James | Powell | |
| WALSALL HEALTHCARE NHS TRUST | | Lisa | Richardson | |
| WALSALL HEALTHCARE NHS TRUST | | Aiden | Plant | |
| WARRINGTON AND HALTON TEACHING HOSPITALS NHS FOUNDATION TRUST | | Zaman | Qazzafi | |
| WARRINGTON AND HALTON TEACHING HOSPITALS NHS FOUNDATION TRUST | | Lisa | Ditchfield | |
| WEST SUFFOLK NHS FOUNDATION TRUST | | Margaret | Moody | |
| WEST SUFFOLK NHS FOUNDATION TRUST | | Veronica | Mendez Moro | |
| WESTERN HEALTH & SOCIAL CARE TRUST | | Tracy | Donaghy | |
| WESTERN HEALTH & SOCIAL CARE TRUST | | Maurice | O'Kane | |
| WESTERN SUSSEX HOSPITALS NHS FOUNDATION TRUST | | R | Sierra | |
| WHITTINGTON HEALTH NHS TRUST | | Chetan | Parmar | |
| WHITTINGTON HEALTH NHS TRUST | | Philippa | Kemsley | |
| WIRRAL UNIVERSITY TEACHING HOSPITAL NHS FOUNDATION TRUST | | David | Harvey | |
| WIRRAL UNIVERSITY TEACHING HOSPITAL NHS FOUNDATION TRUST | | Y | Huang | |
| WYE VALLEY NHS TRUST | | Lisa | Robinson | |
| YEOVIL DISTRICT HOSPITAL NHS FOUNDATION TRUST | | Sarah | Board | |
| YEOVIL DISTRICT HOSPITAL NHS FOUNDATION TRUST | | Andrew | Broadley | |
| YORK TEACHING HOSPITAL NHS FOUNDATION TRUST | | Claire | Brookes | |
| YORK TEACHING HOSPITAL NHS FOUNDATION TRUST | | Neil | Todd | |
| **SIREN Associated Studies and collaborators** | **First name** | | | **Surname** |
| Protective Immunity from T cells to Covid-19 in Health workers (PITCH) | Susanna | | | Dunachie |
| Protective Immunity from T cells to Covid-19 in Health workers (PITCH) | Paul | | | Klenerman |
| Protective Immunity from T cells to Covid-19 in Health workers (PITCH) | Chris | | | Duncan |
| Protective Immunity from T cells to Covid-19 in Health workers (PITCH) | Lance | | | Turtle |
| Protective Immunity from T cells to Covid-19 in Health workers (PITCH) | Alex | | | Richter |
| Protective Immunity from T cells to Covid-19 in Health workers (PITCH) | Thushan | | | De Silva |
| Protective Immunity from T cells to Covid-19 in Health workers (PITCH) | Eleanor | | | Barnes |
| Protective Immunity from T cells to Covid-19 in Health workers (PITCH) | Daniel | | | Wootton |
| Protective Immunity from T cells to Covid-19 in Health workers (PITCH) | Christopher | | | Duncan |
| Protective Immunity from T cells to Covid-19 in Health workers (PITCH) | Rebecca | | | Payne |
| The Humoral Immune Correlates for COVID-19 (HICC) consortium | Jonathan | | | Heeney |
| The Humoral Immune Correlates for COVID-19 (HICC) consortium | Helen | | | Baxendale |
| The Humoral Immune Correlates for COVID-19 (HICC) consortium | Javier | | | Castillo-Olivares |
| The Francis Crick Institute | Rupert | | | Beale |
| The Francis Crick Institute | Edward | | | Carr |
| The Francis Crick Institute | Mary | | | Wu |
| World Influenza Centre, The Francis Crick Institute | Nicola | | | Lewis |
| World Influenza Centre, The Francis Crick Institute | Ruth | | | Harvey |
| Genotype2Phenotype (G2P) | Wendy | | | Barclay |
| Genotype2Phenotype (G2P) | Maya | | | Moshe |
| Genotype2Phenotype (G2P) | Massimo | | | Palmarini |
| Genotype2Phenotype (G2P) | Brian | | | Willett |
| GenOMICC | John Kenneth | | | Baillie |
| British Society for Immunology | Jennie | | | Evans |
| British Society for Immunology | Erika | | | Aquino |
| Wellcome Sanger Institute | Ewan | | | Harrison |
| Wellcome Sanger Institute | Katie | | | Bell |
| Wellcome Sanger Institute | Ya-Lin | | | Huang |
| Wellcome Sanger Institute | Marissa | | | Knoll |
| **Public Health Agencies** | **First name** | | | **Surname** |
| UK Health Security Agency | Susan | | | Hopkins |
| UK Health Security Agency | Victoria | | | Hall |
| UK Health Security Agency | Jasmin | | | Islam |
| UK Health Security Agency | Ana | | | Atti |
| UK Health Security Agency | Omoyeni | | | Adebiyi |
| UK Health Security Agency | Nick | | | Andrews |
| UK Health Security Agency | Hannah | | | Emmett |
| UK Health Security Agency | Jonathan | | | Broad |
| UK Health Security Agency | Nish | | | Kapirial |
| UK Health Security Agency | Simone | | | Dyer |
| UK Health Security Agency | Sophie | | | Russell |
| UK Health Security Agency | Colin | | | Brown |
| UK Health Security Agency | Joanna | | | Conneely |
| UK Health Security Agency | Paul | | | Conneely |
| UK Health Security Agency | Sarah | | | Foulkes |
| UK Health Security Agency | Nabila | | | Fowles-Gutierrez |
| UK Health Security Agency | Nipunadi | | | Hettiarachchi |
| UK Health Security Agency | Jameel | | | Khawam |
| UK Health Security Agency | Edward | | | Monk |
| UK Health Security Agency | Katie | | | Munro |
| UK Health Security Agency | Andrew | | | Taylor-Kerr |
| UK Health Security Agency | Jean | | | Timeyin |
| UK Health Security Agency | Edgar | | | Wellington |
| UK Health Security Agency | Angela | | | Dunne |
| UK Health Security Agency | Dominic | | | Sparkes |
| UK Health Security Agency | Naomi | | | Platt |
| UK Health Security Agency | Anna | | | Howells |
| UK Health Security Agency | Enemona | | | Adaji |
| UK Health Security Agency | Omolola | | | Akinbami |
| UK Health Security Agency | Palak | | | Joshi |
| UK Health Security Agency | Paola | | | Barbero |
| UK Health Security Agency | Meera | | | Chand |
| UK Health Security Agency | Andre | | | Charlett |
| UK Health Security Agency | Michelle | | | Cole |
| UK Health Security Agency | Claire | | | Neill |
| UK Health Security Agency | Anne-Marie | | | O’Connell |
| UK Health Security Agency | Ferdinando | | | Insalata |
| UK Health Security Agency | Tim | | | Brooks |
| UK Health Security Agency | Maria | | | Zambon |
| UK Health Security Agency | Mary | | | Ramsay |
| UK Health Security Agency | Ayoub | | | Saei |
| UK Health Security Agency | Ezra | | | Linley |
| UK Health Security Agency | Simon | | | Tonge |
| UK Health Security Agency | Ashley | | | Otter |
| UK Health Security Agency | Silvia | | | D’Arcangelo |
| UK Health Security Agency | Cathy | | | Rowe |
| UK Health Security Agency | Amanda | | | Semper |
| UK Health Security Agency | Eileen | | | Gallagher |
| UK Health Security Agency | Robert | | | Kyffin |
| UK Health Security Agency | Kate | | | Howell |
| UK Health Security Agency | Jacqueline | | | Hewson |
| UK Health Security Agency | Iain | | | Milligan |
| UK Health Security Agency | Noshin | | | Sajedi |
| UK Health Security Agency | Davina | | | Calbraith |
| UK Health Security Agency | Caio | | | Tranquillini |
| UK Health Security Agency | Jerry | | | Ye Aung Kyaw |
| UK Health Security Agency | Sarah | | | Wallace |
| UK Health Security Agency | Yrene | | | Themistocleous |
| UK Health Security Agency | Blanche | | | Oguti |
| UK Health Security Agency | Sakib | | | Rokadiya |
| UK Health Security Agency | Hannah | | | Emmett |
| Public Health Agency Northern Ireland | Dianne | | | Corrigan |
| Public Health Agency Northern Ireland | Lisa | | | Cromey |
| Glasgow Caledonian University & Public Health Scotland | Lesley | | | Price |
| Glasgow Caledonian University & Public Health Scotland | Nicole | | | Sergenson |
| Glasgow Caledonian University & Public Health Scotland | Sally | | | Stewart |
| Glasgow Caledonian University & Public Health Scotland | Lynne | | | Haahr |
| Glasgow Caledonian University & Public Health Scotland | Desy | | | Nuryunarsih |
| Glasgow Caledonian University | Annelysse | | | Jorgenson |
| Glasgow Caledonian University | Ayodeji | | | Matuluko |
| Glasgow Caledonian University | Melanie | | | Dembinsky |
| Glasgow Caledonian University | Desmond | | | Areghan |
| Glasgow Caledonian University | Alexander | | | Olaoye |
| Public Health Scotland | Josie | | | Evans |
| Public Health Scotland | Jennifer | | | Bishop |
| Public Health Scotland | Jennifer | | | Weir |
| Public Health Scotland | Laura | | | Dobbie |
| Public Health Scotland | Andrew | | | Telfer |
| Public Health Scotland | David | | | Goldberg |
| University of St Andrews | David | | | Crossman |
| Public Health Scotland | Caitlin | | | Plank |
| Public Health Scotland | Laura | | | Naismith |
| Public Health Wales | Ellen | | | De Lacy |
| Public Health Wales | Guy | | | Stevens |
| Public Health Wales | Susannah | | | Froude |
| Public Health Wales | Linda | | | Tyson |
| Health and Care Research Wales | Yvette | | | Ellis |
| Health and Care Research Wales | Chris | | | Norman |
